# Urban tick exposure on Staten Island is higher in pet owners

**DOI:** 10.1371/journal.pone.0311891

**Published:** 2024-11-14

**Authors:** Noriko Tamari, Kacey C. Ernst, Aaron J. Enriquez, Maria A. Diuk-Wasser, Maria P. Fernandez, Kevin Berry, Mary H. Hayden

**Affiliations:** 1 Department of Epidemiology and Biostatistics, College of Public Health, University of Arizona, Tucson, Arizona, United States of America; 2 U.S. Geological Survey, Fort Collins Science Center, Fort Collins, Colorado, United States of America; 3 Department of Ecology, Evolution and Environmental Biology, Columbia University, New York, NY, United States of America; 4 Paul G. Allen School for Global Health, Washington State University, Pullman, WA, United States of America; 5 Department of Economics, University of Alaska Anchorage, Anchorage, Alaska, United States of America; 6 Lyda Hill Institute for Human Resilience, University of Colorado, Colorado Springs, Colorado, United States of America; University of Kentucky College of Medicine, UNITED STATES OF AMERICA

## Abstract

Over the past decade, Lyme and other tick-borne diseases have expanded into urban areas, including Staten Island, New York. While Lyme disease is often researched with a focus on human risk, domestic pets are also at risk of contracting the disease. The present study aims to describe differences in tick exposure, knowledge, attitude, and practices (KAP) between pet owners and non-owners, and to understand preventive strategies practiced by pet owners for themselves and their pets. We conducted KAP surveys via phone in 2020 and via face-to-face interviews in 2021, and we analyzed unique responses from 364 households on Staten Island. Pet owners were more likely to have ever found a tick on themselves or their household members (63%) than non-owners (46%) (p<0.001). Among pet owners, those who owned dogs (dog-only or both dog and cat owners) were more likely to have ever found a tick on their pets than cat-only owners (p<0.001). Compared with non-pet owners, pet owners were more likely both to know that ticks transmit Lyme disease (p<0.001) and to avoid gardening to reduce their tick exposure (p = 0.032), but they were less likely to wear protective clothing or adjust clothing (p = 0.013). Compared with cat owners who had never found a tick on their cats, cat owners who had ever found a tick on their cats were more likely to let their cats go outside (p<0.001). However, reported preventive measures on cats did not differ between pet owners who did and did not report tick exposure. The results indicate that encouraging pet owners to engage in preventative measures, both to protect themselves and their pets, is a potential avenue for healthcare providers and veterinarians to reduce risks from ticks.

## Introduction

Over the past decade, Lyme and other tick-borne diseases have expanded into urban areas, including Staten Island, a borough of New York City (NYC) coextensive with Richmond County, New York [1]. The incidence of Lyme disease per 100,000 was estimated at 10.8 in Richmond County between 2019–2021, which is less than half of the 34.8 per 100,000 incidence in the state of New York during that same time period [2]. However, ticks were found in one-third of yards in 2018, 2019 and 2021 within a half mile of green spaces across Staten Island [3]. Species identified included blacklegged ticks (*Ixodes scapularis*), lone star ticks (*Amblyomma americanum*), and Asian longhorned ticks (*Haemaphysalis longicornis)*; *I*. *scapularis* transmits the agents of Lyme disease, human babesiosis, and anaplasmosis [3, 4]. The population density of tick species in sampled yards on Staten Island increased from 2020 to 2021, with blacklegged ticks increasing from 0.2 to 0.4 ticks/100m^2^, lone star ticks from 0.3 to 0.9 ticks/100m^2^, and Asian longhorned ticks from 1.0 to 4.0 ticks/100m^2^ [4].

While tick-borne diseases are often researched with a focus on human risk, domestic pets, particularly dogs, are also at risk of contracting Lyme disease. Lyme disease in dogs is generally milder and less frequent than in humans, and it is rare in cats [5, 6]. Sentinel surveillance using dogs has been proposed in multiple contexts [7–10], but the relationship between having pets in a household and risk of tick exposure and/or Lyme disease in humans is poorly defined. Jones et al. [11] conducted a study of pet ownership (hereinafter referred as “dog or cat owners”) in high Lyme disease incidence areas which indicated that individuals who own pets have more frequent tick encounters but similar risk of tick-borne diseases compared to individuals without pets. However, increased frequency of tick exposure is associated with increased tick-borne disease risk in other studies [12].

We hypothesized that pet owners may spend more time in recreational and natural areas including tick habitats than non-pet owners and may differ from non-pet owners in the types of preventive activities that their households take to reduce tick exposure (i.e., tick encounters or interactions with environments where ticks occur) on individuals and their pets. Further, we hypothesized that preventive measures may vary between dog and cat owners. For example, this could be due to dog owners perceiving themselves as having higher exposure to the outdoors due to spending time outside playing with or walking their dogs, on their properties and elsewhere. Alternatively, they may be concerned about their dogs bringing ticks in from the outside. Cat owners may not perceive these same risks, especially if they have indoor-only cats. To date, there has been limited research on how tick exposure and preventive measures differ between pet owners and non-owners. Understanding knowledge, attitudes, and practices (KAP) around tick prevention for pets and their owners may provide insights for educational messaging, particularly in areas where tick-borne diseases are newly emerging, and where people are less familiar with the risk factors for tick-borne disease. The present study was conducted on Staten Island, where the population density of tick species has recently increased in yards and parks [1, 3]. The aims were to: 1) determine if tick exposure differs between pet owners and non-owners on Staten Island, 2) describe differences in KAPs between pet owners and non-pet owners, 3) understand preventive strategies for pets practiced by pet owners, and 4) determine if these preventive strategies vary by self-reported tick exposure on individuals and their pets.

## Materials and methods

### Study area and target population

The study area was on Staten Island (149 km^2^), which is the least populated of the five NYC boroughs (Bronx, Brooklyn, Manhattan, Queens, and Staten Island), with a population of 495,747 in 2020 [13]. Of 712 identified cases with Lyme disease in NYC in 2019, 48 cases were confirmed on Staten Island, which was less than the 284 cases in Brooklyn and the 243 cases in Manhattan [14]. Transmission of tick-borne disease agents has occurred locally on Staten Island, while most cases in Brooklyn and Manhattan were contracted during travel outside of NYC [14, 15].

Blacklegged ticks, the primary vectors of the agents of Lyme disease, human babesiosis and anaplasmosis, are spread across Staten Island [14]. Lone star ticks and Asian longhorned ticks are also present; no human pathogens have been found in these ticks on Staten Island [15]. Although American dog ticks are found in all five NYC boroughs, blacklegged tick, lone star ticks and Asian longhorned ticks were found only on Staten Island and in the North Bronx. Staten Island is a newly emerging area for tick-borne diseases, and regular tick monitoring in NYC primarily takes place in its parks [1, 15].

We conducted KAP surveys via phone from June 8, 2020 to August 19 in 2020, due to the COVID-19 pandemic, and via face-to-face interviews from May 20, 2021 to July 20, 2021. Because exposure to ticks in urban areas is restricted to urban greenspaces and residents around these areas, our target ‘at-risk’ populations were residents living in a house with a yard within 500m of parks on Staten Island. The presence of ticks in the parks was verified by performing tick sampling [1, 3]. We focused our sampling on populations living and recreating in residential areas around urban parks because our focus was on local exposure; other urban residents could be exposed by long distance travel, which was beyond the scope of our study.

### Data collection

For the 2020 phone surveys, phone lists for Staten Island were purchased from InfoUSA, Inc. (https://www.infousa.com/) and all households located within 500m of parks where ticks had been found in prior research investigations were eligible to be surveyed. In 2021, we carried out the survey at households located within 500m of park edges on Staten Island as described previously [3]. Briefly, we employed a random cluster sampling strategy and actively recruited 10–15 residents per cluster from randomly selected starting points. We also passively recruited residents through targeted advertising in newspapers and online platforms [3].

The KAP survey included demographics (age, self-reported race/ethnicity, gender, and education), tick exposure history (ever had Lyme disease, personally know anybody who has had Lyme disease, and ever found a tick on individuals or pets), perception on Lyme disease and tick-transmitted diseases (believe Lyme disease on Staten is increasing, and perception of how serious a problem are tick-transmitted diseases on Staten Island), knowledge about diseases that ticks can transmit, and prevention practices to reduce tick exposure on individuals and pets (self/body protection, actions related to avoiding a place/activity to reduce tick exposure on individuals during peak tick season, spending time in parks/natural areas on Staten Island in the past two weeks, gardening/working/playing in a yard in the last two weeks, living environment for pets, products used for pets, and whether or not pet owners let pets go outside) (S1 Appendix).

Verbal consent script was used to introduce the study to potential participants, and verbal consent was subsequently obtained from all participants. For consent, we checked a box on the survey once the participant had consented to proceed to the survey, and we stated that the participant could discontinue at any time. The survey was deemed minimal risk and had ethical clearance from The Institutional Review Board of Columbia University (protocol: AAAS9019).

### Variables

Respondents’ ages were categorized as under 40, 40–49, 50–59, 60–69, 70 and above. Some variables were also recategorized due to limited data or clear interpretation: race as non-white and white; education level as high school graduate/General Equivalency Diploma (GED) or less, Associate’s/Some college/no degree, Bachelor’s degree, and Graduate or professional degree; and perception of how serious a problem are tick-transmitted diseases on Staten Island as very/extremely, slightly/somewhat, not at all, and not sure. For spending time in parks/natural areas or gardening/working/playing in a yard in the past two weeks, the response “parks are closed due to COVID19” was categorized as never.

Individual tick-preventive measures were classified into self/body protection and actions related to avoiding a place/activity to reduce tick exposure on individuals during peak tick season. Self/body protection included applying tick repellent, wearing protective clothes or treated clothing (long pants/ tuck pants into socks), checking for ticks and taking a shower after being outdoors. Actions related to avoiding a place/activity included avoiding going to parks, avoid engaging in outdoor activities in spring/summer/fall, sitting in yards, gardening, playing with kids outdoors, and areas with tick habitats.

### Data analyses

Descriptive tables were stratified by non-pet owners versus pet owners, as well as by dog owners (“dog-only” or “dog and cat” owners) versus cat-only owners, using self-reported tick exposure status on individuals (yes/no), their dogs (yes/no), and their cats (yes/no). We categorized individuals who owned both dogs and cats as dog owners because those with a dog are expected to go outside more to walk the dog, as opposed to those with only cats not necessarily needing to go outside more. Additionally, dogs may bring ticks indoors, which would not be a concern with cats that predominantly stay in the house. We conducted chi-squared tests or Fisher’s exact tests for binary and categorical variables, and t-tests for continuous variables to investigate how demographics, knowledge, attitudes, and practices vary by pet ownership status and self-reported tick exposure status on individuals and their pets. Statistical significance was assessed using 2-sided tests with p-values less than 0.05 considered significant. R (version 4.3.2) was used for all data analyses [16].

## Results

We pooled survey data from 2020 (n = 103) and 2021 (n = 262). After excluding one household due to missing information on pet ownership (n = 1), we had unique responses from 364 households. Although the survey was conducted via phone in 2020 and via face-to-face interviews in 2021, no significant differences were observed in demographics between survey years 2020 and 2021 (S1 Table). Of 201 households who owned a dog or cat, 123 had only dogs, 50 had only cats, and 28 had both dogs and cats.

### Demographics

Pet owners (mean: 53 years, sd:14) were younger than non-pet owners (mean: 58 years, sd:17) (t_287_ = 2.6, p = 0.011). While there were no significant differences in gender and education-level between pet owners and non-pet owners, significant differences were observed in race (*X*^*2*^ = 4.0, *df* = 1, p = 0.046), with white respondents being more likely to own pets than all other respondents. Hispanic or Latino respondents comprised 7.5% of pet owners and 4.3% of non-pet owners (*X*^*2*^ = 1.6, *df* = 1, p = 0.213) (Table 1).

**Table 1:** Characteristics of dog or cat ownership in relation to tick-bite prevention attitudes and actions from a sample of at-risk Staten Island residents.

Variable^1^	Dog/Cat Ownership, n = 364		p-value	Dog/Cat owner, n = 201		p-value
	No	Yes		Dog-only/ dog and cat^a^	Cat-only	
	n = 163	n = 201		n = 151	n = 50	
***Age*** (y), mean (SD)	57.5 (16.8)	53.1 (14.0)	0.011^b^	52.1 (14.0)	56.4 (13.8)	0.067^b^
***Age*** (y)			0.003^c^			0.262^c^
<40	26 (17.4)	34 (17.9)		27 (18.9)	7 (14.9)	
40–49	25 (16.8)	34 (17.9)		26 (18.2)	8 (17.0)	
50–59	22 (14.8)	54 (28.4)		45 (31.5)	9 (19.1)	
60–69	33 (22.1)	42 (22.1)		28 (19.6)	14 (29.8)	
70 and above	43 (28.9)	26 (13.7)		17 (11.9)	9 (19.1)	
Prefer not to say/No answer	14	11		8	3	
***Race***			0.046^c^			0.568^d^
Non-white	25 (17.2)	18 (9.8)		15 (10.9)	3 (6.5)	
White	120 (82.8)	166 (90.2)		123 (89.1)	43 (93.5)	
No answer	18	17		13	4	
***Ethnicity***			0.213^c^			0.421^d^
Non-Hispanic/Latino	135 (92.5)	177 (95.7)		133 (96.4)	44 (93.6)	
Hispanic/Latino	11 (7.5)	8 (4.3)		5 (3.6)	3 (6.4)	
No answer	17	16		13	3	
***Gender***			0.558^d^			1.000^d^
Female	91 (58.3)	124 (63.9)		92 (63.4)	32 (65.3)	
Male	63 (40.4)	68 (35.1)		51 (35.2)	17 (34.7)	
Other	2 (1.2)	2 (1.0)		2 (1.4)	0	
Prefer not to say/No answer	7	7		6	1	
***Education***			0.906^c^			0.807^d^
High school graduate/GED or less	22 (14.3)	28 (14.7)		22 (15.3)	6 (13.0)	
Associate’s, Some college, no degree	41 (26.6)	44 (23.2)		31 (21.5)	13 (28.5)	
Bachelor’s degree	42 (27.3)	55 (28.9)		43 (29.9)	12 (26.1)	
Graduate or professional degree	49 (31.8)	63 (33.2)		48 (33.3)	15 (32.6)	
Prefer not to say	9	11		7	4	
***Have ever had Lyme disease***			0.838^c^			0.767^d^
No	148 (90.8)	181 (91.4)		137 (90.7)	44 (93.6)	
Yes	15 (9.2)	17 (8.6)		14 (9.3)	3 (6.4)	
No answer	0	3		0	3	
***Personally know anybody who has had Lyme disease***			0.410^c^			0.943^c^
No	46 (28.7)	49 (24.9)		37 (25.0)	12 (24.5)	
Yes	114 (71.2)	148 (75.2)		111 (75.0)	37 (75.5)	
No answer	3	4		3	1	
***Believe Lyme disease on Staten is increasing***			0.971^c^			0.522^d^
Increased	109 (66.9)	132 (65.7)		98 (64.9)	34 (68.0)	
About the same	14 (8.6)	17 (8.5)		15 (9.9)	2 (4.0)	
Decreased	5 (3.1)	8 (4.0)		5 (3.3)	3 (6.0)	
Do not know	35 (21.5)	44 (21.9)		33 (21.9)	11 (22.0)	
***How serious a problem are tick-transmitted diseases on Staten Island***			0.327^c^			0.493^d^
Very/extremely	111 (68.1)	127 (63.2)		98 (64.9)	29 (58.0)	
Slightly/somewhat	35 (21.5)	55 (27.4)		41 (27.2)	14 (28.0)	
Not at all	11 (6.7)	12 (6.0)		7 (4.6)	5 (10.0)	
Do not know	6 (3.7)	7 (3.5)		5 (3.3)	2 (4.0)	
Know deer tick/blacklegged tick transmits Lyme disease	93 (57.1)	130 (64.7)	0.138^c^	96 (63.6)	34 (68.0)	0.571^c^
Know ticks can transmit Lyme disease	136 (83.4)	191 (95.0)	<0.001^c^	142 (94.0)	49 (98.0)	0.456^d^
Know ticks can transmit babesiosis	11 (6.7)	16 (8.0)	0.661^c^	10 (6.6)	6 (12.0)	0.235^d^
Know ticks can transmit anaplasmosis	0	4 (2.0)	0.131^d^	2 (1.3)	2 (4.0)	0.259^d^
Know ticks can transmit ehrlichiosis	2 (1.2)	7 (3.5)	0.196^d^	4 (2.6)	3 (6.0)	0.369^d^
Know ticks can transmit alpha-gal syndrome	1 (0.6)	3 (1.5)	0.631^d^	0	3 (6.0)	0.015^d^
Know ticks can transmit encephalitis	1 (0.6)	0	0.448^d^	0	0	-
Know ticks can transmit RMSF	18 (11.1)	31 (15.4)	0.223^c^	18 (11.9)	13 (26.0)	0.017^d^
***Ever found a tick on oneself or household members***			<0.001^c^			0.415^c^
No	88 (54.3)	74 (36.8)		58 (38.4)	16 (32.0)	
Yes	74 (45.7)	127 (63.2)		93 (61.6)	34 (68.0)	
No answer	1	0		0	0	
***Ever found a tick on their pet***						<0.001^c^
No	-	110 (55.6)	-	69 (46.3)	41 (83.7)	
Yes	-	88 (44.4)	-	80 (53.7)	8 (16.3)	
No answer	-	3	-	2	1	
***Self/body protection***						
Apply tick repellent	52 (31.9)	82 (40.8)	0.080^c^	61 (40.4)	21 (42.0)	0.842^c^
Wear protective clothes/adjust clothing^e^	70 (42.9)	61 (30.3)	0.013^c^	43 (28.5)	18 (36.0)	0.316^c^
Wear treated clothing	10 (6.1)	11 (5.5)	0.788^c^	8 (5.3)	3 (6.0)	1.000^d^
Check for ticks	31 (19.0)	52 (25.9)	0.121^c^	43 (28.5)	9 (18.0)	0.143^c^
Shower/bathe after being outdoors	6 (3.7)	13 (6.5)	0.235^c^	7 (4.6)	6 (12.0)	0.093^d^
At least one self/body protection	109 (66.9)	138 (68.7)	0.701^c^	103 (68.2)	35 (70.0)	0.813^c^
***Actions related to avoiding a place/activity to reduce tick exposure on individuals during peak tick season***						
Avoid going to parks	36 (22.1)	44 (21.9)	0.964^c^	35 (23.2)	9 (18.0)	0.443^c^
Avoid outdoor activities in spring, summer, and fall	20 (12.3)	19 (9.5)	0.388^c^	16 (10.6)	3 (6.0)	0.415^d^
Avoid sitting in yards	3 (1.8)	8 (4.0)	0.358^d^	7 (4.6)	1 (2.0)	0.682^d^
Avoid gardening	9 (5.5)	3 (1.5)	0.032^c^	2 (1.3)	1 (2.0)	1.000^d^
Avoid playing with kids outdoors	2 (1.2)	7 (3.5)	0.196^d^	6 (4.0)	1 (2.0)	0.683^d^
Avoid tick habitat	28 (17.2)	46 (22.9)	0.179^c^	35 (23.2)	11 (22.0)	0.864^c^
At least one action related to avoiding a place/activity	65 (39.9)	93 (46.3)	0.221^c^	72 (47.7)	21 (42.0)	0.485^c^
At least one tick preventive measure^f^	122 (74.8)	160 (79.6)	0.280^c^	121 (80.1)	39 (78.0)	0.746^c^
***Spent time in parks/natural areas on Staten Island in the last two weeks*** ^g^			0.300^c^			0.572^c^
Never	75 (46.3)	85 (42.7)		63 (42.3)	22 (44.0)	
At least once	40 (24.7)	40 (20.1)		29 (19.5)	11 (22.0)	
At least 3 days a week	17 (10.5)	21 (10.6)		14 (9.4)	7 (14.0)	
Most days	30 (18.5)	53 (26.6)		43 (28.9)	10 (20.0)	
Not sure	1	2		2	0	
***Gardening*, *working or playing in your yard in the last two weeks*** ^g^			0.768^c^			0.939^c^
Never	23 (14.4)	27 (13.4)		19 (12.6)	8 (16.0)	
At least once	24 (15.0)	30 (14.9)		23 (15.2)	7 (14.0)	
At least 3 days a week	25 (15.6)	40 (19.9)		30 (19.9)	10 (20.0)	
Most days	88 (55.0)	104 (51.7)		79 (52.3)	25 (50.0)	
Not sure	3	0		0	0	

Abbreviation: GED, General Equivalency Diploma; RMSF, Rocky Mountain Spotted Fever

Notes:

Values are the number of individuals, with percentages reported in parentheses. Some percentages may not add up to exactly 100% due to rounding. “Prefer not to say”, “No answer”, or “Not sure” responses are excluded when calculating percentages and conducting statistical tests.

^a^ Only dog (n = 123), both dog and cat (n = 28).

^b^ T-test.

^c^ Pearson’s Chi square test.

^d^ Fisher’s exact test due to expected counts being less than five.

^e^ “Adjust clothing” means “long pants/ tuck pants into socks.”

^f^ At least one measure of self/body protection and/or actions related to avoiding a place/activity.

^g^ “At least once” was defined as “at least once, but less than three times.” “Most time” means “more than 3 days a week.”

### Tick exposure

Pet owners were more likely to have ever found a tick on themselves or their household members (63%) than non-owners (46%) (*X*^*2*^ = 11.1, *df* = 1, p<0.001). Among pet owners, those who owned dogs (dog-only or both dog and cat owners) were more likely to have ever found a tick on their pets than cat-only owners (*X*^*2*^ = 20.1, *df* = 1, p<0.001) (Table 1).

### Knowledge

Pet owners were more likely to know that ticks transmit Lyme disease (95% vs. 83%; *X*^*2*^ = 13.2, *df* = 1, p<0.001). However, 65% of pet owners and 57% of non-owners knew specifically that deer tick/blacklegged tick transmits Lyme disease (*X*^*2*^ = 2.2, *df* = 1, p = 0.138). While 15% of pet owners and 11% of non-owners were aware that Rocky Mountain Spotted Fever (RMSF) is a tick-transmitted disease, fewer than 5% in either group were familiar with other tick-borne diseases (Table 1).

### Tick preventive measures/outdoor activities

Pet owners were less likely to wear protective clothing or adjust clothing and avoid gardening to reduce their tick exposure during peak tick season compared with non-pet owners (protective/adjusting clothing: 30% for pet owners, 43% for non-pet owners, *X*^*2*^ = 6.2, *df* = 1, p = 0.013; avoid gardening: 2% for pet owners, 6% for non-pet owners, *X*^*2*^ = 4.6, *df* = 1, p = 0.032). Applying tick repellent to individuals and wearing protective clothes/adjusting clothing were the most reported tick preventive strategies for pet owners and non-owners, respectively. Tick checks were performed by 26% of pet owners and 19% of non-pet owners. Very few people practiced avoidance strategies such as avoiding sitting in the yard, gardening, or playing with children outdoors in either group (< 10%). Further, recreation in parks and natural areas was similar between groups (*X*^*2*^ = 3.7, *df* = 3.0, p = 0.300) (Table 1). Of those who never spent time in parks/natural areas on Staten Island in the last two weeks, two respondents stated that the parks were closed due to COVID19.

### Living environment and preventive measures taken on pets

Most dogs went outdoors (96%), but only 31% of cats did. Using at least one product to prevent ticks on pets was reported by 46% of pet owners (63% for dogs, 14% for cats). There were no significant differences in preventive actions taken by pet owners who did and did not report a history of tick exposure on themselves or their household members (Tables 2 and 3). No significant differences were found in preventive actions taken on their pets between dog owners who had ever or never found a tick on their dogs. However, significant differences were observed between cat owners (Table 2). Compared with cat owners who had never found a tick on their cats, cat owners who had ever found a tick were more likely to let their cats go outdoors (Fisher’s exact test, p<0.001) or apply Revolution (spot on) to them for tick prevention (Fisher’s exact test, p = 0.032), whereas they were less likely to prevent cats from going outside (Fisher’s exact test, p = 0.025) (Table 3). While there was a significant difference in using at least one product between cat owners who reported a tick on their cats (39%) and those who did not (13%) (Fisher’s exact test, p = 0.038), no significance was observed in taking at least one tick preventive action for their cats (*X*^*2*^ = 0.2, *df* = 1, p = 0.647).

**Table 2:** Tick-bite preventive measures taken on dogs from a sample of at-risk Staten Island residents.

Variable	Ever found a tick on oneself or household members, n = 151		p-value	Ever found a tick on their dog, n = 149		p-value
	No	Yes		No	Yes	
	n = 58	n = 93		n = 70	n = 79	
***Living environment***						
Dog goes outdoors	54 (93.1)	91 (97.8)	0.204^c^	66 (94.3)	78 (98.7)	0.187^c^
***Product used for their dogs*** ^a^						
Bansect	0	1 (1.1)	1.000^c^	0	1 (1.3)	1.000^c^
Bravecto	4 (6.9)	5 (5.4)	0.733^c^	4 (5.7)	4 (5.1)	1.000^c^
Credelio	0	1 (1.1)	1.000^c^	1 (1.4)	0	0.470^c^
Frontline	15 (25.9)	26 (28.0)	0.778^d^	16 (22.9)	24 (30.4)	0.301^d^
Interceptor	1 (1.7)	1 (1.1)	1.000^c^	2 (2.9)	0	0.219^c^
K9	3 (5.2)	8 (8.6)	0.532^c^	5 (7.1)	6 (7.6)	0.916^c^
NexGard	8 (13.8)	12 (12.8)	0.875^d^	10 (14.3)	10 (12.7)	0.771^d^
Revolution	1 (1.7)	3 (3.2)	1.000^c^	1 (1.4)	3 (3.8)	0.623^c^
Seresto	8 (13.8)	8 (8.6)	0.314^d^	7 (10.0)	9 (11.4)	0.784^d^
Simparica	2 (3.4)	7 (7.5)	0.483^c^	3 (4.3)	6 (7.6)	0.502^c^
Trifexis	1 (1.7)	0	1.000^c^	1 (1.4)	0	0.470^c^
Vectra	0	2 (2.2)	0.524^c^	0	2 (2.5)	0.499^c^
Collar (unknown)	1 (1.7)	4 (4.3)	0.649^c^	3 (4.3)	2 (2.5)	0.666^c^
Oral treatment (unknown)	4 (6.9)	2 (2.2)	0.204^c^	2 (2.9)	4 (5.1)	0.685^c^
Spot-on treatment (unknown)	1 (1.7)	1 (1.1)	1.000^c^	1 (1.4)	1 (1.3)	1.000^c^
At least one product used	46 (79.3)	76 (81.7)	0.715^d^	55 (78.6)	63 (79.5)	0.860^d^
***Action related to avoiding a place***						
Not let dogs go outside	1 (1.7)	2 (2.2)	1.000^c^	2 (2.9)	0	0.219^c^
At least one tick preventive action taken on their dog^d^	46 (79.3)	76 (81.7)	0.715^d^	57 (81.4)	63 (79.5)	0.796^d^

Notes: Values are the number of individuals, with percentages reported in parentheses.

^a^ BRAVECTO, Credelio, Interceptor, NexGard, and Trifexis are oral treatments, Seresto is available as a collar or oral treatment, and the others are spot-on treatments.

^b^ At least one measures among product used for their dogs and action related to avoiding a place.

^c^ Fisher’s exact test due to expected counts being less than five.

^d^ Pearson’s Chi square test.

**Table 3:** Tick-bite preventive measures taken on cats from a sample of at-risk Staten Island residents.

Variable	Ever found a tick on oneself or household members, n = 78		p-value	Ever found a tick on their cats, n = 77		p-value
	No	Yes		No	Yes	
	n = 21	n = 57		n = 64	n = 13	
***Living environment***						
Cat goes outdoors	1 (9.1)	14 (36.8)	0.137^c^	8 (20.0)	7 (87.5)	<0.001^c^
Missingness	10	19		24	5	
***Product used for their cats*** ^a^						
Bravecto	0	1 (1.8)	1.000^c^	1 (1.6)	0	1.000^c^
Frontline	0	2 (3.5)	1.000^c^	2 (3.1)	0	1.000^c^
Revolution	1 (4.8)	4 (7.0)	1.000^c^	2 (3.1)	3 (23.1)	0.032^c^
NexGard	0	1 (1.8)	1.000^c^	1 (1.6)	0	1.000^c^
Seresto	0	2 (3.5)	1.000^c^	2 (3.1)	0	1.000^c^
Sergeant’s Guardian	0	1 (1.8)	1.000^c^	0	1 (7.7)	0.169^c^
Spot-on treatment (unknown)	0	1 (1.8)	1.000^c^	0	1 (7.7)	0.169^c^
At least one product used	1 (4.8)	12 (21.1)	0.167^c^	8 (12.6)	5 (38.5)	0.038^c^
***Action related to avoiding a place***						
Not let cats go outside	8 (38.1)	21 (36.8)	0.919^d^	28 (43.8)	1 (7.7)	0.025^c^
At least one tick preventive action taken on their cats^d^	8 (38.1)	32 (56.1)	0.157^d^	34 (53.1)	6 (46.2)	0.647^d^

Notes: Values are the number of individuals, with percentages reported in parentheses.

Missing data are excluded when calculating percentages and conducting statistical tests.

^a^ BRAVECTO and NexGard are oral treatments, Seresto is available as a collar or oral treatment, and the others are spot-on treatments.

^b^ At least one measures among product used for their cats and action related to avoiding a place.

^c^ Fisher’s exact test due to expected counts <five.

^d^ Pearson’s Chi square test.

## Discussion

Our findings based on a sample of at-risk residents on Staten Island, an urban area with significant green spaces where tick-borne diseases are newly emerging were consistent with previous reports of tick exposure being higher among individuals who own pets [11]. There were several differences in knowledge and practices between pet owners and non-owners, but they do not account for tick exposure variability. Pet owners were more likely to know that ticks could transmit Lyme disease, which could theoretically translate into greater adherence to tick prevention practices. However, pet owners and non-owners engaged in similar levels of personal preventive measures except that wearing protective clothing and avoiding gardening to reduce their tick exposure were more prevalent among non-owners. Our hypothesis that pet owners spend more time in recreational and natural areas that support tick habitat was not supported, as reported outdoor activities were similar for pet owners and non-pet owners. Cat owners who reported finding ticks on their cats were more likely to use Revolution for their cats than those who did not report ticks. Other preventive products used for pets did not differ based on self-reported tick exposure for individuals and their pets.

Pet owners reported less use of protective clothing than non-pet owners as a preventive strategy. Pet owners may feel uncomfortable wearing such clothing due to the different types of exercise they engage in with their pets. Since pet owners generally tend to spend more time outdoors than non-pet owners [17–20], wearing protective or adjusting clothes can make them feel too warm, leading them to avoid this measure. Additionally, the difference in the use of protective or adjusting clothing may be explained by the different distribution in age and ethnicity between pet owners and non-pet owners. In the present study, pet owners were younger and included fewer Hispanic/Latino individuals compared to non-pet owners. Factors related to these demographics, such as the type of outdoor activities, occupation, or income, may influence the use of protective or adjusting clothing. For example, similar to pet owners, younger individuals, who typically engage in more physical activities than older ones [21–23], may opt not to wear such clothing.

In the present study, both pet owners and non-owners spent time in their yard on most days (more than three days a week) and rarely checked for ticks or took a shower or bath after being outdoors, despite recommendations by the Centers for Disease Control and Prevention (CDC) [24] and the effectiveness of these measures in preventing tick-borne diseases [25]. Participation in preventive practices among our sample of at-risk Staten Island residents was relatively low, with 20% performing a tick check and 5% taking a shower or bath after outdoor activities, as compared to 30–70% and 15–40%, respectively, in studies of the general population [26] and 55–68% and 40–59%, respectively, in several Lyme disease-endemic areas in the United States [27–29]. Since ticks, including blacklegged ticks, were found in one-third of yards within a half mile of green spaces across Staten Island [3], such preventative practices are an important potential avenue for both pet owners and non-owners to reduce risk, especially considering factors like the potentially high cost of purchasing protective clothes and the safety concerns around chemical repellents [26, 29]. As demonstrated in previous studies [26, 30], community-wide educational intervention may increase preventive actions taken by individuals both for themselves and their pets.

The present study demonstrated lower tick prevention on pets (46%) and higher tick exposure on individuals and pets (over 60%) on Staten Island compared to several tick-borne disease endemic areas, where tick prevention for pets exceeded 80% and tick exposure was around 30% [11, 31]. The idea that pets may bring ticks into shared living spaces is an area that needs more exploration. We found that most dogs and some cats were reported to go outdoors, though it was not specified in the question whether respondents related this to tick exposure. Assuming that dogs are more likely to spend time outside than cats, this could explain why people who owned a dog were more likely to have found a tick on their pet than cat-only owners. It could be anticipated that pet owners who took precautions with their pets would be less likely to have pets that harbored ticks and more likely to report lower levels of exposure on themselves and household members. Yet, precautions did not significantly differ between respondents or their household members who were exposed to ticks and those who were not.

Owners of cats reporting ticks on their cats were more likely to treat them with Revolution for tick prevention than cat owners who did not report ticks. These cat owners may have started using Revolution after discovering ticks on their cats. Alternatively, because our data did not distinguish between Revolution (selamectin) and Revolution Plus (selamectin and sarolaner), Revolution alone may have been ineffective against ticks, particularly blacklegged ticks [32]. Further investigations with larger sample sizes and more detailed information could yield additional insights. It is possible that some participants did not report products used to prevent ticks on their pets, or that those who used preventive measures engaged in behaviors that exposed them to more tick habitat. The temporality, frequency, dose, and adherence of medical collars or chews/drops on pets were not captured, nor were their efficacy or costs. Their effects wane over time and may be affected by improper dosing [29].

There are several limitations in the present study. First, we did not account for quantities in some questions. The “ever found a tick” questions did not distinct between finding one tick or 100 ticks on oneself or household members, or their pets. Additionally, our data did not include the amount of time spent outdoors when being outside. The information might influence tick preventative measures they take for themselves and their pets. Second, the survey was conducted during the height of the COVID-19 pandemic. Some people altered their behaviors, including frequency and patterns of outdoor activities, which may not fully represent their lifestyles post-pandemic [33–36]. Third, we did not specify the duration of pet ownership. Global interest in pet adoptions temporally increased during the COVID-19 pandemic [37]. Some pet owners might have been new owners who adopted during the COVID-19 pandemic and had not yet modified their behavior. Duration of ownership might influence tick exposure history, knowledge, or behaviors among pet owners. Fourth, the self/body protection actions and place/activity avoidance actions that were used in the present study were the most common preventive measures for individuals in other geographic areas [27–29], but these variables may not cover all preventive measures.

## Conclusion

Pet owners or their household members were exposed to ticks more frequently than non-owners in a sample of at-risk Staten Island residents, an urban area with extensive green spaces. However, reported preventive measures for pets did not differ between pet owners who reported tick exposure and those that did not report tick exposure. The results indicate that healthcare providers and veterinarians could attempt to reduce risks from ticks by encouraging pet owners to engage in preventative measures for themselves and their pets.

We are unable to determine if pet owners might be exposed to higher risk habitats while recreating in natural areas than non-pet owners, whether through their own activities or their pets’ activities. Further investigations into the details of pet, pet owner, and non-pet owner activities in parks or yards, as well as more in-depth assessments of frequencies of administering prevention and control strategies, may yield more insights into the relationship between pets and human exposure to ticks.

## Supporting information

S1 Appendix Survey instrument. (DOCX)

S1 Dataset Anonymized dataset. (XLSX)

S1 Table Demographic and pet ownership characteristics of at-risk Staten Island residents by survey year. (DOCX)

## References

[1] VanAcker MC, Little EA, Molaei G, Bajwa WI, Diuk-Wasser MA. Enhancement of risk for lyme disease by landscape connectivity, New York, New York, USA. Emerg Infect Dis. 2019;25(6):1136. doi: 10.3201/eid2506.181741 31107213 PMC6537717

[2] New York State Department of Health. CHIRS Dashboard 2023 [cited 2024 August 9]. Available from: https://apps.health.ny.gov/public/tabvis/PHIG_Public/chirs/reports/#county.

[3] Gregory N, Fernandez MP, Diuk-Wasser M. Risk of tick-borne pathogen spillover into urban yards in New York City. Parasites & vectors. 2022;15(1):1–14. doi: 10.1186/s13071-022-05416-2 35948911 PMC9365221

[4] Vasan A. 2022 Health Advisory #10: Tick-borne Disease Advisory 2022 [cited 2024 August 9]. Available from: https://www.nyc.gov/assets/doh/downloads/pdf/han/advisory/2022/tick-borne-diseases.pdf.

[5] Little SE, Heise SR, Blagburn BL, Callister SM, Mead PS. Lyme borreliosis in dogs and humans in the USA. Trends in parasitology. 2010;26(4):213–8. doi: 10.1016/j.pt.2010.01.006 20207198

[6] Gibson MD, Young CR, Omran MT, Palma K, Edwards JF, Rawlings JA, et al. Lyme disease in an experimental cat model. Int J Angiol. 1995;4:155–9.

[7] Lindenmayer JM, Marshall D, Onderdonk AB. Dogs as sentinels for Lyme disease in Massachusetts. Am J Public Health. 1991;81(11):1448–55. doi: 10.2105/ajph.81.11.1448 1951802 PMC1405676

[8] Goossens H, Van Den Bogaard A, Nohlmans M. Dogs as sentinels for human Lyme borreliosis in The Netherlands. J Clin Microbiol. 2001;39(3):844–8. doi: 10.1128/JCM.39.3.844-848.2001 11230393 PMC87839

[9] Hamer SA, Tsao JI, Walker ED, Mansfield LS, Foster ES, Hickling GJ. Use of tick surveys and serosurveys to evaluate pet dogs as a sentinel species for emerging Lyme disease. Am J Vet Res. 2009;70(1):49–56. doi: 10.2460/ajvr.70.1.49 19119948

[10] Smith FD, Ballantyne R, Morgan ER, Wall R. Estimating Lyme disease risk using pet dogs as sentinels. Comp Immunol Microbiol Infect Dis. 2012;35(2):163–7. doi: 10.1016/j.cimid.2011.12.009 22257866

[11] Jones E, Hinckley A, Hook S, Meek J, Backenson B, Kugeler K, et al. Pet ownership increases human risk of encountering ticks. Zoonoses Public Health. 2018;65(1):74–9. doi: 10.1111/zph.12369 28631423 PMC7053298

[12] Hook SA, Nawrocki CC, Meek JI, Feldman KA, White JL, Connally NP, et al. Human‐tick encounters as a measure of tickborne disease risk in Lyme disease endemic areas. Zoonoses Public Health. 2021;68(5):384–92. doi: 10.1111/zph.12810 33554467 PMC10883354

[13] U.S. Census Bureau. Populations and People ‐ Staten Island borough, Richmond County, New York 2020 [cited 2024 July 31]. Available from: https://data.census.gov/profile/Staten_Island_borough,_Richmond_County,_New_York?g=060XX00US3608570915.

[14] New York City Department of Health and Mental Hygiene. Lyme Disease in New York City 2020 [cited 2024 July 31]. Available from: https://www.nyc.gov/assets/doh/downloads/pdf/zoo/lyme-disease-nyc-2020.pdf.

[15] New York City Department of Health and Mental Hygiene. Tick-borne Diseases Other Than Lyme Disease in New York City 2020 [cited 2024 July 31]. Available from: https://www.nyc.gov/assets/doh/downloads/pdf/zoo/tick-borne-diseases-report-2020.pdf.

[16] R Core Team. R: A language and environment for statistical computing. R foundation for statistical computing Vienna, Austria; 2024.

[17] Mein G, Grant R. A cross-sectional exploratory analysis between pet ownership, sleep, exercise, health and neighbourhood perceptions: the Whitehall II cohort study. BMC Geriatr. 2018;18:1–9. 30092763 10.1186/s12877-018-0867-3 PMC6085675

[18] Dembicki D, Anderson J. Pet ownership may be a factor in improved health of the elderly. J Nutr Elder. 1996;15(3):15–31. doi: 10.1300/J052v15n03_02 8948954

[19] Cutt H, Giles-Corti B, Knuiman M, Timperio A, Bull F. Understanding dog owners’ increased levels of physical activity: results from RESIDE. Am J Public Health. 2008;98(1):66–9. doi: 10.2105/AJPH.2006.103499 18048786 PMC2156050

[20] Martins CF, Soares JP, Cortinhas A, Silva L, Cardoso L, Pires MA, et al. Pet’s influence on humans’ daily physical activity and mental health: a meta-analysis. Frontiers in Public Health. 2023;11:1196199. doi: 10.3389/fpubh.2023.1196199 37325330 PMC10262044

[21] Elgaddal N, Kramarow EA, Reuben C. Physical activity among adults aged 18 and over: United States, 2020. Hyattsville, MD: National Center for Health Statistics; 2022. 36043905

[22] Katzmarzyk PT, Lee I-M, Martin CK, Blair SN. Epidemiology of physical activity and exercise training in the United States. Prog Cardiovasc Dis. 2017;60(1):3–10. doi: 10.1016/j.pcad.2017.01.004 28089610

[23] Hawkins MS, Storti KL, Richardson CR, King WC, Strath SJ, Holleman RG, et al. Objectively measured physical activity of USA adults by sex, age, and racial/ethnic groups: a cross-sectional study. International Journal of Behavioral Nutrition and Physical Activity. 2009;6:1–7. 19493347 10.1186/1479-5868-6-31 PMC2701914

[24] Centers for Disease Control and Prevention. Preventing tick bites 2024 [cited 2024 August 9]. Available from: https://www.cdc.gov/ticks/prevention/.

[25] Connally NP, Durante AJ, Yousey-Hindes KM, Meek JI, Nelson RS, Heimer R. Peridomestic Lyme disease prevention: results of a population-based case–control study. Am J Prev Med. 2009;37(3):201–6. doi: 10.1016/j.amepre.2009.04.026 19595558

[26] Eisen L. Personal protection measures to prevent tick bites in the United States: knowledge gaps, challenges, and opportunities. Ticks Tick Borne Dis. 2022;13(4):101944. doi: 10.1016/j.ttbdis.2022.101944 35364518 PMC10859966

[27] Beck A, Bjork J, Biggerstaff BJ, Eisen L, Eisen R, Foster E, et al. Knowledge, attitudes, and behaviors regarding tick-borne disease prevention in Lyme disease-endemic areas of the Upper Midwest, United States. Ticks Tick Borne Dis. 2022;13(3):101925. doi: 10.1016/j.ttbdis.2022.101925 35255349 PMC10947721

[28] Butler AD, Sedghi T, Petrini JR, Ahmadi R. Tick-borne disease preventive practices and perceptions in an endemic area. Ticks Tick Borne Dis. 2016;7(2):331–7. doi: 10.1016/j.ttbdis.2015.12.003 26704290

[29] Niesobecki S, Hansen A, Rutz H, Mehta S, Feldman K, Meek J, et al. Knowledge, attitudes, and behaviors regarding tick-borne disease prevention in endemic areas. Ticks Tick Borne Dis. 2019;10(6):101264. doi: 10.1016/j.ttbdis.2019.07.008 31431351 PMC10948045

[30] Malouin R, Winch P, Leontsini E, Glass G, Simon D, Hayes EB, et al. Longitudinal evaluation of an educational intervention for preventing tick bites in an area with endemic lyme disease in Baltimore County, Maryland. Am J Epidemiol. 2003;157(11):1039–51. doi: 10.1093/aje/kwg076 12777368

[31] de Wet S, Rutz H, Hinckley AF, Hook SA, Campbell S, Feldman KA. Love the ones you’re with: Characteristics and behaviour of Maryland pets and their owners in relation to tick encounters. Zoonoses Public Health. 2020;67(8):876–81. doi: 10.1111/zph.12768 33112510

[32] Vatta AF, Young DR, Everett WR, King VL, Cherni JA, von Reitzenstein M, et al. Efficacy of a new topical formulation containing selamectin plus sarolaner against three common tick species infesting cats in the United States. Vet Parasitol. 2019;270:S19–S25. doi: 10.1016/j.vetpar.2018.10.013 30470637

[33] Brand R, Timme S, Nosrat S. When pandemic hits: Exercise frequency and subjective well-being during COVID-19 pandemic. Front Psychol. 2020:2391. doi: 10.3389/fpsyg.2020.570567 33071902 PMC7541696

[34] Doubleday A, Choe Y, Busch Isaksen T, Miles S, Errett NA. How did outdoor biking and walking change during COVID-19?: A case study of three US cities. PLoS One. 2021;16(1):e0245514. 33471858 10.1371/journal.pone.0245514 PMC7816985

[35] Alba C, Pan B, Yin J, Rice WL, Mitra P, Lin MS, et al. COVID-19’s impact on visitation behavior to US national parks from communities of color: evidence from mobile phone data. Sci Rep. 2022;12(1):13398. doi: 10.1038/s41598-022-16330-z 35927271 PMC9352905

[36] Taff BD, Rice WL, Lawhon B, Newman P. Who started, stopped, and continued participating in outdoor recreation during the COVID-19 pandemic in the United States? Results from a national panel study. Land. 2021;10(12):1396.

[37] Ho J, Hussain S, Sparagano O. Did the COVID-19 pandemic spark a public interest in pet adoption? Frontiers in Veterinary Science. 2021;8:647308. doi: 10.3389/fvets.2021.647308 34046443 PMC8145284

