# Supplementary material for: Urban tick exposure on Staten Island is higher in pet owners
Source: PLoS One. 2024 Nov 14;19(11):e0311891. doi: 10.1371/journal.pone.0311891 (PMC11563360; doi:10.1371/journal.pone.0311891)
Supplement: S1 Appendix — (DOCX) [file pone.0311891.s001.docx]

**S1 Appendix. Survey instrument.**

| **Question** | **Response** | |
| --- | --- | --- |
| Would you mind telling me how old you are? | Age:  ☐ Prefer not to say  ☐ No answer | |
| How would you classify yourself in regard to race? *(check all that apply)* | ☐ American Indian/Alaskan Native  ☐ Asian/Pacific-Islander  ☐ Black/African American  ☐ White  ☐ Multi-racial  ☐ Other  ☐ No answer | |
| Do you consider yourself Hispanic or Latino? | ☐ Yes  ☐ No  ☐ No answer | |
| And your gender? | ☐ Male  ☐ Female  ☐ Other  ☐ Prefer not to say  ☐ No answer | |
| What is the highest level of education you have completed? | ☐ no formal education  ☐ Less than 9th grade  ☐ 9th to 12th  ☐ High school graduate/GED  ☐ Some college, no degree  ☐ Associate’s degree  ☐ Bachelor’s degree  ☐ Graduate or professional degree  ☐ No answer | |
| Have you had Lyme disease? | ☐ Yes  ☐ No  ☐ No answer | |
| Do you personally know anybody else who has had Lyme disease? | ☐ Yes  ☐ No  ☐ No answer | |
| Do you think the number of Lyme disease cases on Staten Island has increased, decreased or hasn’t changed much in the last 5 years? | ☐ Increased  ☐ About the same  ☐ Decreased  ☐ No answer  ☐ I don’t know | |
| When you think about other health concerns that you might have (besides COVID-19), how serious a problem are tick-transmitted diseases on Staten Island on a scale of 1 to 5 - 1 being not at all serious and 5 being extremely serious? *(Read the options if needed and check their answer by reading it back to them)* | ☐ 5: Extremely serious  ☐ 4: Very serious  ☐ 3: Somewhat serious  ☐ 2: Slightly serious  ☐ 1: Not at all serious  ☐ Not sure  ☐ No answer | |
| Do you know what kind of tick transmits Lyme  disease? *(Don’t read. Mark all mentioned)* | ☐ Deer tick/blacklegged tick  ☐ Lone star  ☐ Dog ticks  ☐ All ticks  ☐ Only small ones  ☐ I don’t know  ☐ No answer  ☐ Other: | |
| Could you tell me which diseases ticks can transmit? *(Don’t read. Mark all* *mentioned)* | ☐ I don’t know  ☐ Lyme  ☐ Babesiosis  ☐ Anaplasmosis  ☐ Ehrlichiosis  ☐ No answer  ☐ Other: | |
| Have you ever found a tick on you or a member of your household? | ☐ No  ☐ Yes  ☐ No answer | |
| Have you ever found a tick on your pet? | ☐ Yes - dog(s)  ☐ Yes - cat(s)  ☐ No  ☐ No answer | |
| How do you protect yourself from tick bites? *(Don’t read. Mark all mentioned)* | ☐ Tick repellent  ☐ Adjust clothing (long pants/ tuck  pants into socks)  ☐ Wear treated clothing (ex. Permethrin treated pants)  ☐ Check for ticks  ☐ Shower or bathe after being outdoors  ☐ Avoid tick habitat  ☐ Other: | |
| What activities, if any, have you stopped or avoided to reduce your tick exposure during peak tick season?  *(Don’t read. Mark all mentioned)* | ☐ Gardening  ☐ Going to parks  ☐ Sitting in yards  ☐ Playing with kids outdoors  ☐ Outdoor activities in spring, summer and fall  ☐ None, I don’t do anything differently  ☐ Other: | |
| In the last two weeks, how many days have you visited any parks/natural areas on Staten Island?  *(Read the options)* | ☐ Most days  ☐ At least three days a week  ☐ At least once (but less than 3)  ☐ Never  ☐ Parks are closed due to COVID19  ☐ Not sure  ☐ No answer | |
| In the last two weeks, how many days did you spend  gardening, working or playing in your yard?  *(Read the options)* | ☐ Most days  ☐ At least three days a week  ☐ At least once (but less than 3)  ☐ Never  ☐ Not sure  ☐ No answer | |
| Does your dog/cat go outdoors? | Dog | Cat |
|  | ☐ Yes  ☐ No | ☐ Yes  ☐ No |
| How do you prevent ticks on your pet? *(Don’t read the options and mark all that are used)* | Dog | Cat |
|  | ☐ I don’t let them go outside  ☐ Bravecto (oral)  ☐ Frontline (spot on)  ☐ K9 Advantix (spot on)  ☐ Revolution  (spot on)  ☐ NexGard (oral)  ☐ Seresto (collar)  ☐ Vectra (spot on)  ☐ Other | ☐ I don’t let them go outside  ☐ Bravecto (oral)  ☐ Frontline (spot on)  ☐ K9 Advantix (spot on)  ☐ Revolution (spot on)  ☐ NexGard (oral)  ☐ Seresto (collar)  ☐ Vectra (spot on)  ☐ Other |
