# Supplementary material for: Urban tick exposure on Staten Island is higher in pet owners
Source: PLoS One. 2024 Nov 14;19(11):e0311891. doi: 10.1371/journal.pone.0311891 (PMC11563360; doi:10.1371/journal.pone.0311891)
Supplement: S1 Table — (DOCX) [file pone.0311891.s003.docx]

**S1 Table. Demographic and pet ownership characteristics of at-risk Staten Island residents by survey year.**

| **Variable**^1^ | **Year of survey, n=364** | | **p-value** |
| --- | --- | --- | --- |
|  | **2020** | **2021** |  |
|  | **n=103** | **n=261** |  |
| ***Age*** (y), mean (SD) | 54.9 (14.5) | 55.1 (15.8) | 0.909^a^ |
| ***Age*** (y) |  |  | 0.409^b^ |
| <40 | 16 (16.2) | 44 (18.3) |  |
| 40-49 | 15 (15.2) | 44 (18.3) |  |
| 50-59 | 25 (25.3) | 51 (21.2) |  |
| 60-69 | 27 (27.3) | 48 (20.0) |  |
| 70 and above | 16 (16.2) | 53 (22.1) |  |
| Prefer not to say/No answer | 4 | 21 |  |
| ***Race*** |  |  | 0.461^b^ |
| Non-white | 10 (10.9) | 33 (13.9) |  |
| White race | 82 (89.1) | 204 (86.1) |  |
| No answer | 11 | 24 |  |
| ***Ethnicity*** |  |  | 0.799^b^ |
| Non-Hispanic/Latino | 90 (93.8) | 222 (94.5) |  |
| Hispanic/Latino | 6 (6.2) | 13 (5.5) |  |
| No answer | 7 | 26 |  |
| ***Gender*** |  |  | 0.576^c^ |
| Female | 62 (60.8) | 153 (61.7) |  |
| Male | 40 (39.2) | 91 (36.7) |  |
| Other | 0 | 4 (1.6) |  |
| Prefer not to say/No answer | 1 | 13 |  |
| ***Education*** |  |  | 0.643^b^ |
| High school graduate/GED or less | 15 (15.3) | 35 (14.2) |  |
| Associate’s, Some college, no degree | 22 (22.4) | 63 (25.6) |  |
| Bachelor's degree | 32 (32.7) | 65 (26.4) |  |
| Graduate or professional degree | 29 (29.6) | 83 (33.7) |  |
| Prefer not to say | 5 | 15 |  |
| ***Dog/Cat Ownership*** |  |  | 0.465^b^ |
| No | 43 (41.7) | 120 (46.0) |  |
| Yes | 60 (58.3) | 141 (54.0) |  |
| **Dog/Cat owner** |  |  | 0.460^b^ |
| Dog-only/ dog and cat^d^ | 43 (71.7) | 108 (76.6) |  |
| Cat only | 17 (28.3) | 33 (23.4) |  |
| NA (non-pet owner) | 43 | 120 |  |

*Abbreviation: GED, General Equivalency Diploma; NA, not applicable*

*Notes:*

*Values are the number of individuals, with percentages reported in parentheses. Some percentages may not add up to exactly 100% due to rounding. “Prefer not to say” or “No answer” responses are excluded when calculating percentages and conducting statistical tests.*

*^a^ T-test.*

*^b^ Pearson’s Chi square test.*

*^c^ Fisher’s exact test due to expected counts being less than five.*

*^d^ Only dog (n=123), both dog and cat (n=28).*
